# Translational utility of rodent hippocampal auditory gating in schizophrenia research: a review and evaluation

**DOI:** 10.1038/tp.2015.77

**Published:** 2015-06-23

**Authors:** J Smucny, K E Stevens, A Olincy, J R Tregellas

**Affiliations:** 1 Neuroscience Program, University of Colorado Anschutz Medical Campus, Aurora, CO, USA; 2 Research Service, Denver VA Medical Center, Denver, CO, USA; 3 Department of Psychiatry, University of Colorado Anschutz Medical Campus, Aurora, CO, USA

## Abstract

Impaired gating of the auditory evoked P50 potential is one of the most pharmacologically well-characterized features of schizophrenia. This deficit is most commonly modeled in rodents by implanted electrode recordings from the hippocampus of the rodent analog of the P50, the P20–N40. The validity and effectiveness of this tool, however, has not been systematically reviewed. Here, we summarize findings from studies that have examined the effects of pharmacologic modulation on gating of the rodent hippocampal P20–N40 and the human P50. We show that drug effects on the P20–N40 are highly predictive of human effects across similar dose ranges. Furthermore, mental status (for example, anesthetized vs alert) does not appear to diminish the predictive capacity of these recordings. We then discuss hypothesized neuropharmacologic mechanisms that may underlie gating effects for each drug studied. Overall, this review supports continued use of hippocampal P20–N40 gating as a translational tool for schizophrenia research.

## Introduction

Development of translational assays that accurately predict drug response from animal models to human patients is one of the most pressing needs for research in psychiatric diseases, including schizophrenia. The purpose of these assays is to provide low-cost screening tools for investigational compounds to increase the probability of success for new drugs as they proceed through the drug development pipeline. Indeed, high failure rates for investigational compounds (95% or greater for neuropsychiatric diseases)^1^ has led to many pharmaceutical companies either downsizing or dropping research platforms altogether,^2^ highlighting the need for an effective translational toolbox.

Schizophrenia research has been plagued by problems in developing animal models that predict clinical efficacy, in large part due to the fact that no single animal model is able to recapitulate the complex symptomatology of schizophrenia. Researchers have therefore attempted to develop models that show abnormalities in its neurophysiological features. These include deficits in prepulse inhibition,^3^ neuronal synchrony^4^ and auditory (P50) gating. P50 gating deficits are among the most thoroughly examined features of schizophrenia, with well-studied genetic bases (for example, reduced nicotinic α7 receptor signaling)^5, 6^ and many studies examining the effects of therapeutic manipulation. Importantly, P50 gating has also been extensively studied in the rodent using implanted electrode recordings in the hippocampus. Accordingly, this review includes an introduction to P50 gating in schizophrenia, followed by an evaluation of the translational predictive power of studying this electrophysiological deficit.

## Schizophrenia and P50 gating

The study of P50 gating impairment in schizophrenia has its origins from work in the 1960s by McGhie and Chapman^7^ as well as Venables,^8^ who published extensive patient case reports of perceptual abnormalities. Many of these reports described a ‘hypervigilant' state in which patients were unable to ignore persistent distracting noises in the environment. As a result, patients found it hard to concentrate on any one stimulus in a noisy environment, such as the voice of a single person in a bustling crowd. Hypervigilance was later hypothesized to have a role in the emergence of positive symptoms. For example, increased salience of the sounds of squealing tires may cause the noises to be reinterpreted as a screaming baby.^9^

**Figure 1:**
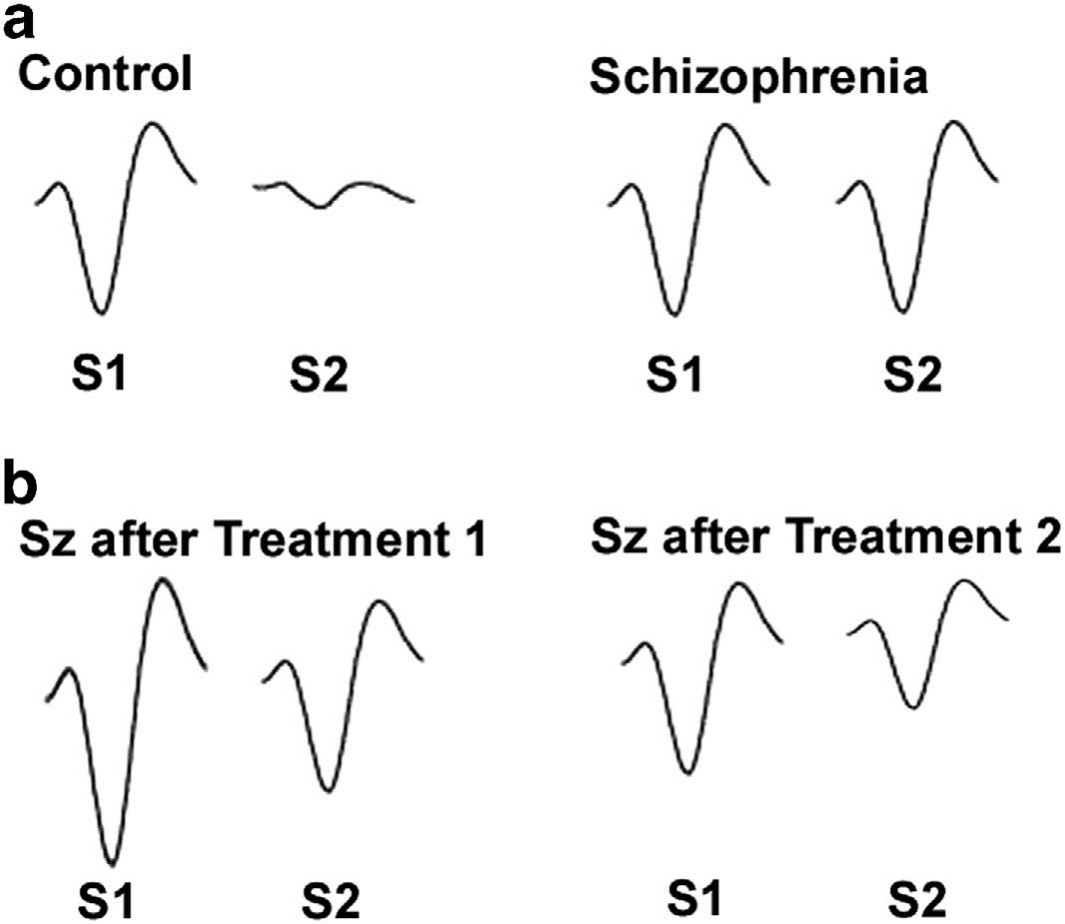
Representative P50 event-related potentials illustrating P50 gating deficits in schizophrenia. (**a**) In a healthy subject (left pair of traces), the brain inhibits its response to the second (S2) of a pair of repeated stimuli. A patient with schizophrenia (right pair of traces) is unable to inhibit response to this stimulus. (**b**) This effect can be normalized by treatments that increase response to the first stimulus (S1, left pair of traces) or decrease response to second stimulus (S2, right pair of traces). S1, first stimulus; S2, second stimulus; SZ, schizophrenia.

The ‘hypervigilant' state found in schizophrenia led Adler *et al.*^10^ to postulate that patients may show a deficit in the ability of the brain to physiologically decrease, or ‘gate,' its response to repeated stimuli. This brain response is postulated to have a major role in the ability of healthy subjects to subconsciously ignore irrelevant, incessant stimuli in the environment such as a clock ticking.^9^ On the basis of electroencephalographic methods developed in the 1960s for studying repetitive auditory stimuli,^11^ Adler *et al.*^10^ observed reduced capacity in schizophrenia to diminish early (50 ms post stimulus, or P50) responses to the second of a pair closely spaced identical (~0.5 s) clicks (Figure 1a). This phenomenon has since been replicated in many laboratories, is predictive of cognitive function in several domains including attention^12, 13, 14, 15^ and is one of the most frequently investigated electrophysiological phenotypes in schizophrenia. The relationships between P50 gating and positive and negative symptomatology are unclear and an important area for future investigation (reviewed by Potter *et al.*^13^). As discussed later in this review, studies have found that this phenotype can be normalized by a number of compounds, either by increasing the physiological response to the first click (relative to the second) and/or decreasing the response to the second click (relative to the first; Figure 1b).

**Figure 2:**
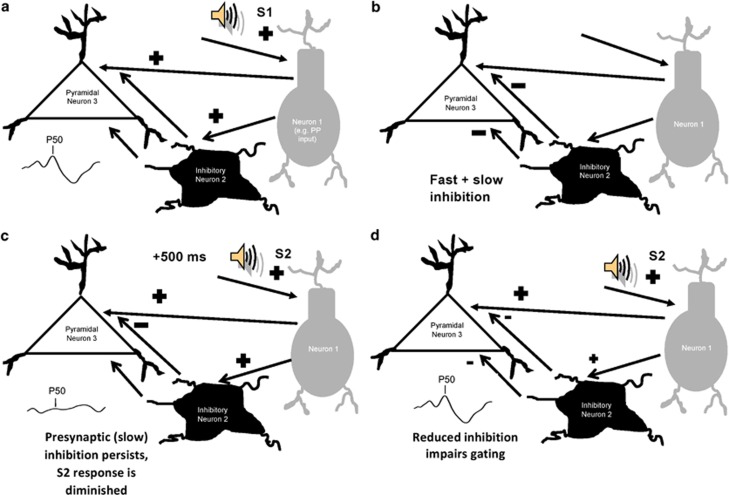
Cartoon schematic of the hypothesized neuronal circuit responsible for sensory gating and its deficits in schizophrenia. Waveform positive polarity is upwards. (**a**) In a healthy subject, a sound stimulus excites Neuron 1 (for example, the perforant path (PP) input to the hippocampus), which in turn excites hippocampal pyramidal Neuron 3. Neuron 1 also excites inhibitory Neuron 2. (**b**) Neuron 2 reduces glutamate release by Neuron 1 via activation of presynaptic GABA-B receptors (slow inhibition) as well as inhibits Neuron 3 via activation of postsynaptic GABA-A receptors (fast inhibition). (**c**) Step 3: a second sound stimulus arrives 500 ms later and excites Neuron 1. Unlike the previous stimulus, Neuron 1 cannot excite Neuron 3 owing to persistent (slow) inhibition from Neuron 2. Signal from the second stimulus is, therefore, reduced or ‘gated.' (**d**) In a patient with schizophrenia, gating deficits may arise from reduced GABAergic signaling caused by dysfunction of Neuron 2.

A remarkable aspect of P50 gating is the simplicity behind the neuronal circuitry that may underlie the phenomenon.^16^ In its most basic form, this process can be accomplished with only three neurons: two excitatory neurons and an intermediate inhibitory neuron (Figure 2). In the paired-click paradigm, the first, ‘conditioning' stimulus (S1) excites Neuron 1, which in turn excites inhibitory Neuron 2 and excitatory pyramidal Neuron 3 (Figure 2a). Activation of Neuron 2, in turn, induces release of the inhibitory neurotransmitter GABA. GABA release causes fast inhibition of Neuron 3 via postsynaptic GABA-A receptors as well as slow, persistent inhibition of glutamate release onto Neuron 3 from Neuron 1 (via presynaptic GABA-B receptors^17^). Persistent inhibition in particular diminishes the response of Neuron 3 for up to 8 s (Figure 2b).^9^ Consequently, if the second, ‘test' stimulus (S2) arrives <1 s after S1, S2 event-related potential amplitude is reduced compared with S1 (Figure 2c). A reduction of the ability of Neuron 2 to modulate this circuit (for example, by reduced α7 nicotinic receptor expression on inhibitory Neuron 2) is postulated to underlie gating deficits in schizophrenia (Figure 2d).^16^ These deficits are maximal when stimuli are spaced 0.5 s apart, as typically presented in sensory gating paradigms.^10, 18^

## Modeling P50 gating in the rodent hippocampus

The first human and animal studies examined P50 gating and its rodent analog (the P20, N40 and P20–N40) from cortical surface recordings at the center of the skull or vertex.^10, 19^ Although this technique provided a straightforward method of measuring gating in single subjects, it did not provide information as to where gating occurs in the brain. The first attempt at localizing gating was conducted in anesthetized rats using depth electrodes.^20^ This study found that the hippocampus CA3 subfield, relative other areas in the auditory processing pathway (medial geniculate, auditory cortex) showed robust gating that was sensitive to amphetamine-induced impairment, consistent with previous findings in rats using a cortical surface electrode.^19^

**Figure 3:**
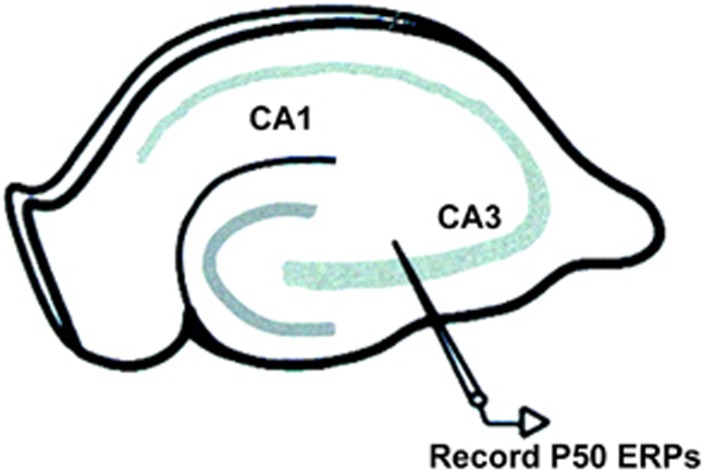
Location of CA3 electrodes in the mouse hippocampus for recording P20/N40 evoked potentials. Figure adapted from Guo *et al.*^24^ ERP, event-related potential.

The results of this study became the basis for using depth electrode recordings from the hippocampus (CA3 region, Figure 3) to study auditory gating in mouse and rat models of schizophrenia. Hippocampal localization of gating and its schizophrenia-associated deficit in humans has since been supported in studies using implanted electrodes from epileptic patients^21^ and noninvasive techniques such as electroencephalography combined with functional magnetic resonance imaging.^22, 23^

**Figure 4:**
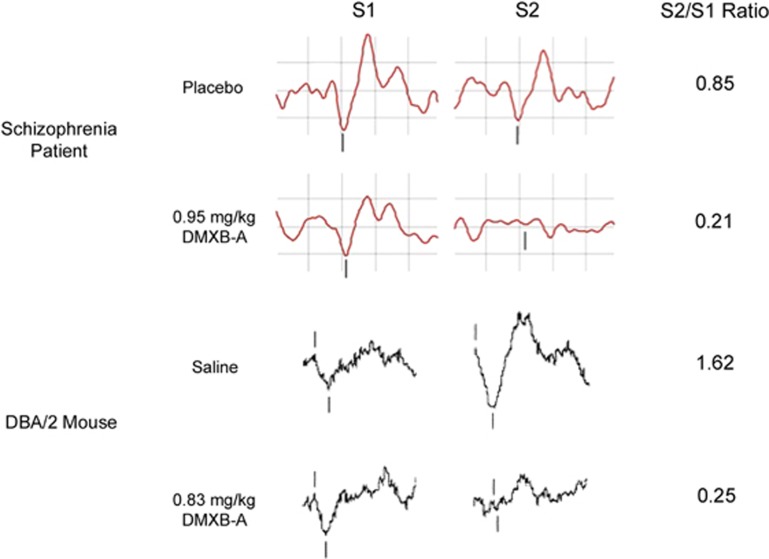
Comparison of S1 waveforms, S2 waveforms and S2/S1 ratios during placebo/saline and DMXB-A administration as measured by EEG in a schizophrenia patient (adapted from Olincy *et al.*^25^) and an implanted electrode in the CA3 subfield of the DBA/2 mouse hippocampus (adapted from Simosky *et al.*^26^). Positive polarity is downwards. Vertical hash marks denote the P50 in the patient and the P20–N40 in the mouse. Similar improvements on sensory gating were observed after DMXB-A treatment in both patients and mice. DMXB-A, 3-2,4 dimethoxybenzylidene anabaseine; EEG, electroencephalography.

Representative P50 and P20–N40 waveforms taken from the vertex electrode of a human subject and from an electrode implanted in the hippocampus of a mouse (respectively) are shown in Figure 4.

## Hippocampal gating as a translational tool: a review and evaluation

Although hippocampal depth recordings are the most commonly used method for examining auditory gating in the rodent, methodological discrepancies between animal and human P50 gating studies challenge the translational utility of the technique. The majority of rodent studies differ from human studies in regard to mental state (anesthetized in rodents vs awake in patients), treatment duration (acute in rodents vs chronic in patients) and route of drug administration (intraperitoneal/subcutaneous in rodents vs oral in patients; see Methodological effects and considerations). The hippocampal location of the electrode is also disputed, as functional imaging studies in humans and animals have found many gating generators in addition to the hippocampus.

To that end, the following sections evaluate hippocampal auditory gating as a translational tool in schizophrenia research. The primary measure of interest is the correspondence between drug effects on rodent hippocampal gating and human P50 gating. As part of this analysis, we examine the qualitative effects of drug dose and anesthesia, and focus on gating (the ratio of S2 to S1 amplitude) as well as the relative contribution of changes in S1 and S2 amplitude to drug effects. As the focus of this review is schizophrenia, we primarily report on studies that examined rodent models of schizophrenia (see Box 1) and human patients with the disease. These studies are grouped by drug and summarized in Table 1a–c.

### Correspondence between rodent and human studies

As a whole, hippocampal findings from rodent models matched very well with scalp recordings of P50 gating in human subjects (Table 1a–c). Across similar dose ranges, drugs generally had comparable effects on not only gating, but also S1 and S2 amplitudes. For example, 3-2,4 dimethoxybenzylidene anabaseine (DMXB-A) had remarkably consistent effects on gating that showed a similar dose-dependent mechanism (S2-driven) across species. Mental status, frequency of dosing or route of drug administration did not qualitatively alter the overall effect or dose dependence on these results. Although some discrepancies were noted, we believe that they may be readily explained by differences in the pharmacologic background and/or dose(s) used between studies. Comparisons between rodent and human findings for each drug are summarized in the following sections, which have been divided into three subsections based on their primary pharmacologic mechanisms of action during gating paradigms: nicotinic, dopaminergic and serotonergic.

### Nicotinic-based treatments—nicotine, varenicline, DMXB-A, tropisetron, donepezil, perinatal choline

Interest in nicotinic receptor-based treatments for schizophrenia is primarily due to high rates (70–80%) of nicotine self-administration (for example, cigarette smoking) in patients compared with the general population (20–30%).^83, 84^ Smoking patients also consume more cigarettes and intake more nicotine on average than other smokers.^85^ Patients are hypothesized to smoke for several reasons, including (1) having ‘nothing better to do'^84^ as a consequence of being unemployed or otherwise leading an inactive lifestyle, (2) relieving dopaminergic inhibition caused by antipsychotic medication and (3) ‘self-medicating' in an attempt to correct an intrinsic deficit in nicotinic signaling.^84, 86^

The latter hypothesis has gained the most traction to date. Acute nicotine administration reverses sensory processing abnormalities in schizophrenia, including deficits in prepulse inhibition,^87^ eye tracking^88, 89, 90, 91^ and P50 gating (see below). Genetic studies have linked deficits in P50 gating in the illness to polymorphisms on the α7 nicotine receptor gene promoter and a partial duplication of the α7 gene, possibly contributing to receptor expression deficits observed in the illness.^92, 93, 94, 95^ The other highly expressed central nervous system nicotinic receptor subtype, α4β2, is also abnormally regulated in schizophrenia smokers.^96^

In regard to P50 gating, early studies in schizophrenia patients focused on nicotine, a high affinity agonist of α4β2 and low affinity agonist of α7 receptors.^97^ From a gating perspective, nicotine may be expected to affect both S1 and S2 amplitude due to its ability to activate α4β2 receptors on pyramidal cells and α7 receptors on inhibitory interneurons, respectively. Nicotine may also influence P50 amplitudes by increasing catecholamine (dopamine and NE) release in the hippocampus.^98^ Previous studies, however, only partially support this hypothesis, as some studies show S1-driven effects on gating^43, 45, 46^ and others show S2 (or S1 and S2)-driven effects^35, 44, 47^ (Table 1a). Studies in patients suggest that nicotine transiently improves gating by decreasing S2 amplitude,^47^ with effects reversing (or not observed) after 30 min of nicotine deprivation.^47, 48, 49^ We suggest that inconsistencies in these results may be explained by differences in pharmacologic background between studies. Experiments in which nicotine did not increase S1 amplitude were conducted in the presence of antipsychotic medication (mostly first-generation drugs).^35, 47^ A consistent effect of first-generation antipsychotics is increased S1 response (Table 1b), possibly owing to differences in excitability induced by dopaminergic blockade. It is possible that in the presence of these drugs, S1 has already been increased to its maximal amount, precluding any further increase by nicotine.

Similar to nicotine, varenicline activates both α4β2 and α7 receptors, where it acts as a partial agonist on the α4β2 subtype and full agonist on the α7 subtype.^99^ Its primary indication is for smoking cessation,^100^ as the drug is able to potentiate nicotinic signaling while minimally inducing the nicotine-induced dopamine release associated with addiction.^100^ In regard to auditory gating and schizophrenia, the drug has mixed effects (Table 1a). In DBA/2 mice, the drug improves gating by decreasing S2 amplitude at low doses and increasing S1 amplitude at higher doses.^50^ The dose dependence of varenicline may be due to the observation that α7 receptors desensitize as a function of increasing agonist concentration,^97^ reducing S2-mediated effects at high doses of drug. A pilot human study using a ‘low' dose (0.012 mg kg^−1^), however, showed no significant effects of acute varenicline administration on gating.^51^ This negative finding may be due to the low sample size (*n*=6) of the study, as it had to be ended prematurely due to side effects. A later clinical trial in a larger sample of patients showed improved gating after chronic administration of the drug (0.013 mg kg^−1^).^52^ Consistent with the mouse finding near this dose, the effect was driven by a reduction in S2 amplitude. Similar to their effects in nicotine studies, antipsychotic medications may have also occluded any effects of varenicline on S1 amplitude in this study.

To date, the majority of investigational research and development into targeting the cholinergic system in schizophrenia has focused on compounds that activate the α7 receptor. The most thoroughly investigated α7 compound in P50 gating and other studies in schizophrenia is DXMB-A (GTS-21), an α7 nicotinic receptor partial agonist and α4β2 antagonist.^101^ Tropisetron, a partial α7 receptor agonist and 5-HT(3) receptor antagonist, has also been investigated using P50 gating paradigms in animals and human patients.

As demonstrated in Table 1a, our review of DMXB-A and tropisetron studies demonstrated remarkably consistent effects in both animals and humans, where the drug reliably improved gating by decreasing S2 amplitude under similar dose ranges (Figure 4).^25, 26, 44, 53, 54, 55, 56, 57, 58^ Tropisetron also improved gating, an effect driven by increasing S1 amplitude.^55, 58^ DMXB-A was effective when administered chronically, despite concerns that prolonged use may induce receptor desensitization.^54^ A study that investigates the effects of chronic DMXB-A on gating in smoking schizophrenia patients is currently ongoing.

Decreased S2 amplitude-driven P50 gating improvement after DMXB-A administration is consistent with its pharmacology, as activation of the α7 receptor preferentially increases nicotinic current on inhibitory interneurons over pyramidal cells in the hippocampus^102^ (Figure 2). The dual effect (S1 and S2) observed with tropisetron may be due to its antagonism of 5-HT(3) receptors. Blockade of these serotonin receptors may relieve tonic inhibition of hippocampal acetylcholine release,^103^ consequently increasing activation of both α7 receptors on inhibitory neurons (decreasing S2 amplitude) as well as α4β2 receptors on excitatory pyramidal cells (increasing S1 amplitude; Figure 2).

Another strategy for potentiating nicotinic signaling is to increase ACh levels by inhibiting its degradation by enzymes. The acetylcholinesterase inhibitor donepezil (Aricept) is one such compound. Its primary indication is for the treatment of Alzheimer's, as the disease is characterized by the pervasive loss of cholinergic neurons.^104^ Research into its effects in schizophrenia was motivated by studies showing a loss of cholinergic (muscarinic and nicotinic) receptor expression in schizophrenia^94, 95, 105, 106, 107, 108^ as well as a negative correlation between choline acetyltransferase (the enzyme that synthesizes ACh) activity and cognitive symptom severity.^109^

The effects of donepezil on sensory gating have been investigated in one study in rats and one in schizophrenia patients (Table 1a). Consistent with its hypothesized effects as a generalized cholinergic enhancer (activating α7 and α4β2 nicotinic receptors on inhibitory interneurons and excitatory pyramidal cells), the drug improved gating in rats by both potentiating S1 amplitude and reducing S2 amplitude.^59^ Drug effects did not reach significance in schizophrenia patients (*P*=0.14, in the direction of improved gating).^60^ Unlike the study in rats, this result was driven by a small (nonsignificant) reduction in S2 amplitude in patients. Reasons for the discrepancy may be that (1) the drug was tested in healthy rats and not a schizophrenia model and (2) dose differences (0.43 mg kg^−1^ in rats, 0.13 mg kg^−1^ in patients) between studies. The dose in the schizophrenia patient study was based on doses that have demonstrated efficacy in Alzheimer's while minimizing side effects.^110^

Schizophrenia researchers have primarily focused on treatments for patients already diagnosed with the illness. A complementary strategy is to develop preventative interventions early in development—as soon as the prenatal stage—to minimize risk later in life.^111^ Due to the complexity of schizophrenia and the necessity for measuring a physiological component that precedes diagnosis, Ross and colleagues have focused on an electrophysiological endophenotype of schizophrenia (P50 gating), which is impaired in infants with psychotic parents.^112^

Choline, a dietary precursor of ACh that is found in eggs and red meats, has been shown to increase neurogenesis in adult rats after prenatal administration^113^ as well as hippocampal dendritic arborization and soma size.^114^ Choline is also an important constituent of cell membranes and is therefore particularly important during fetal development when new cell membranes are being rapidly produced.^115, 116, 117, 118^ In addition to inducing ACh synthesis, choline may potentiate nicotinic signaling through its selective agonism of α7 receptors.^119, 120, 121^

On the basis of its activity as a cholinergic potentiator and its role in development, Stevens *et al.*^61^ proposed that choline given to dams would improve P20–N40 gating in their DBA/2 offspring. Consistent with this hypothesis, Stevens *et al.*^61^ found that DBA/2 dams given a supplemental choline diet (5 × normal choline) produced offspring that showed improved gating. The effect was driven by decrease in S2 amplitude (Table 1a). Interestingly, offspring given supplemental choline also demonstrated increased α7 receptor expression, potentially contributing to the gating effect. Ross *et al.*^63^ extended these findings to human studies, demonstrating a similar dose regimen of perinatal choline also improved P50 gating in healthy infants. Akin to mouse studies, the effect was driven by a decrease in S2 amplitude (Table 1a). As this preliminary study was in healthy mothers, future studies may examine the effects of perinatal choline in infant offspring of patients with schizophrenia.

### Dopaminergic-based treatments—haloperidol and amisulpride

As initially postulated, the dopamine hypothesis of schizophrenia states that symptoms arise from hyperactive dopamine transmission.^122^ Indeed, the first antipsychotics (‘first-generation,' or ‘typical') were all dopaminergic receptor antagonists. Furthermore, many drugs that increase dopaminergic transmission, such as amphetamine, induce psychosis in healthy individuals.^30, 123^ This hypothesis has more recently been revised to postulate that positive symptoms, in particular, arise from hyperactivation of the dopaminergic D2 receptor subtype in mesolimbic brain regions.^122^

In regard to P50 gating, the effects of haloperidol (Haldol) and other first-generation (typical) antipsychotics were first examined in the 1980s owing to their widespread use in treating schizophrenia at the time. As summarized in Table 1b, the majority of these and later studies (animal and human) have observed no effect of dopaminergic drugs on gating.^35, 65, 66, 67, 68, 69, 70^ Although the drug does appear to increase S1 amplitude in some studies, it often increases S2 amplitude to a similar extent, resulting in a net no change in gating.^35, 66, 67^ The neurobiological mechanisms that underlie these changes are unclear, but may be related to effects on hippocampal excitability.^66, 124, 125^ The exception to this pattern is in rat studies in which normal gating was perturbed by amphetamine; these experiments showed significant reversal of gating effects by haloperidol.^20, 64^

As a whole, these studies suggest that drugs for which D2 blockade is a primary mechanism of action, such as typical antipsychotics, are unlikely to improve gating deficits in schizophrenia. Consistent with this view, a genetic linkage study in schizophrenia patients found no associations between variation of dopamine receptor genes and P50 gating.^126^ A later study that examined the effects of genetic variation in a dopamine transporter 1 gene found that healthy subjects that may have higher dopamine levels due to hypoexpression of dopamine transporter 1 showed improved gating, suggesting that dopaminergic blockade may actually worsen gating in some individuals.^127^

### Serotonergic-based treatments: clozapine, ondansetron, olanzapine

Beginning with clozapine (Clozaril) in the 1970s, a second class of antipsychotic medications emerged well after the first-generation antipsychotic use became widespread. These drugs, called ‘second-generation' or ‘atypical' antipsychotics, featured the ability to treat positive symptoms while minimizing the extrapyramidal side effects observed with typical antipsychotics. Unlike typical antipsychotics, some atypicals (particularly clozapine) may show small pro-cognitive effects in schizophrenia.^128^

A feature shared by atypical antipsychotics is relatively higher antagonism for serotonin (5-HT) receptors relative to D2 receptors.^128^ In regard to sensory gating, the ability of these drugs to antagonize the 5-HT(3) receptor subtype is hypothesized to have a particularly important role,^65, 71, 72, 73^ as blockade of this receptor may induce ACh release,^103^ increasing activation of nicotinic receptors. Activation of pre and postsynaptic nicotinic receptors on inhibitory neurons may increase release of the inhibitory neurotransmitter GABA onto excitatory neurons (see Figure 2) consequently decreasing S2 amplitude (Figure 1b). Gating may be further improved by increasing S1 amplitude through cholinergic activation of postsynaptic receptors on excitatory pyramidal neurons. Some atypicals, however (such as risperidone), do not improve P50 gating in schizophrenia patients, suggesting that either additional mechanisms account for the pro-gating effects of these drugs or that the effect is dependent on the ability of each drug to affect the activity of various neurotransmitter systems.

Ondansetron (Zofran) is perhaps the pharmacologically ‘cleanest' example of a serotinergic drug with pro-gating effects across animal and human studies. It is not classified as an antipsychotic, as it is a selective 5-HT(3) receptor antagonist and therefore does not block D2 receptors.^129^ Its selectivity for the 5-HT(3) receptor allows researchers to isolate pro-gating effects due to blockade of this particular serotonin receptor subtype.

Consistent with the demonstrated ability of 5-HT(3) receptor blockade to indirectly activate nicotinic receptors by increasing ACh release, ondansetron improved gating in DBA/2 mice^71^ and schizophrenia patients^72^ in a manner similar to nicotinic agonists (Table 1c). These effects were driven by increased S1 amplitude and decreased S2 amplitudes in mice,^71^ but only decreased S2 amplitude in patients.^72^ The dual effect observed in animals may be due to the ability of ondansetron to induce activation of α7 nicotinic receptors on inhibitory interneurons as well as α4β2-receptor on excitatory pyramidal cells (Figure 2). The discrepancy between human and animal studies may be due to the antipsychotic medications that the patients were taking at the time of the study (six of the eight subjects were taking typical antipsychotics), as these drugs have been shown to increase S1 amplitude (Table 1b). A ceiling effect on S1 amplitude may have therefore been reached, preventing any further increase by ondansetron.

Olanzapine (Zyprexa) is a second-generation, atypical antipsychotic medication. Like the majority of atypical antipsychotics, olanzapine is a potent 5-HT receptor antagonist, as well as a low(er) affinity dopamine receptor antagonist.^130^ Consistent with its demonstrated effects on the cholinergic system,^131, 132^ olanzapine has been shown to enhance auditory gating in the DBA/2 mouse^73^ and in a pilot sample of six schizophrenia patients (~50% improvement in P50 suppression; Table 1c).^133^ The patient finding, however, was not replicated in later studies.^68, 69, 74^ This discrepancy may be due to differences in the dose used to examine effects in the mouse vs human patients. Patient studies have examined doses up to 10-fold higher than in the mouse study to maximize clinical stability. Such high doses are necessary to treat positive symptoms in schizophrenia due to the relatively lower binding affinity of olanzapine for D2 receptors compared with 5-HT receptors. High doses, however, may also increase ACh efflux to the extent that they induce nicotinic receptor desensitization, preventing the improvement in gating observed at lower doses. Future studies may examine the pro-gating effects of lower doses on olanzapine in patients treated with an additional antipsychotic.

Another atypical antipsychotic that has been investigated using sensory gating paradigms is clozapine (Clozaril). Clozapine has a complex binding profile. The drug is an antagonist at dopamine and 5-HT receptors, with relatively low potency (compared with typical antipsychotics) at D2 receptors.^134^ Clozapine is also a muscarinic, histaminergic and adrenergic receptor antagonist.^135^

As summarized in Table 1c, in gating studies, clozapine has had variable effects. Most animal and human studies have observed improved gating with the drug,^65, 74, 76, 77, 78, 79, 80, 133^ possibly due to its ability to increase ACh release and activate nicotinic receptors. Two patient studies, in contrast, found no effect of clozapine on gating.^69, 81^ Importantly, in one of these studies, patients showed normal P50 gating, potentially occluding any effect of the drug.^69^ Animal studies have found that low doses of clozapine improve gating by primarily decreasing S2 amplitude.^65, 76^ Higher doses of clozapine, however, may improve gating by both decreasing S2 and increasing S1 amplitudes.^65, 75, 76^ Human studies have mostly reported S1 increases after clozapine administration (Becker *et al.*;^80^ Light *et al.;*^133^ Nagamoto *et al.*;^78^ Nagamoto *et al.*^79^) although one study found that the drug decreased S2 amplitude (Adler *et al.*^74^).

Subtype-specific modulation of S1 and S2 amplitude may help to explain the dose-dependent effects on each waveform by clozapine. The α7 receptors display dose-dependent desensitization, in which high concentrations of agonist (for example, ACh) cause receptor inactivation.^97^ It is possible that a relatively small increase of ACh release (induced by low concentrations of clozapine) may favor α7 channel opening over desensitization, reducing S2 amplitude. The dose-dependent increase of S1, on the other hand, may be due to dopaminergic antagonism observed at higher doses, similar to the effect of typical antipsychotics such as haloperidol.^65, 66, 67^

## Other drugs

In this section, we discuss the effects of other classes of treatments on auditory gating that have not been tested using identical drugs across species and/or evaluated from hippocampal electrode-based rodent recordings, but, nonetheless, support gating as an effective translational tool for schizophrenia research.

### Norepinephrine

Interest in the effects of adrenergic modulation of sensory gating originates from work by Adler *et al.*^19^ who demonstrated that increased arousal/stress associated with new environments impaired gating in rats as measured by cortical surface electrodes. Furthermore, acute administration of amphetamine, which induces NE (and dopamine) release, impairs gating in rats by reducing S1 amplitude.^19^ This effect is reversible by the NE-depleting toxin *N*-2-chloroethyl-*N*-ethyl-2-bromobenzylamine (DSP4).^136^

Following this initial characterization, later studies focused on examining the specific receptor basis for NE effects. NE acts on two main groups of receptors (α and β adrenergic) each of which has several subtypes that vary in nervous system expression patterns, subcellular (pre vs postsynaptic) localization and affinity for NE.^137, 138, 139, 140^ An early cortical surface electrode study by Stevens *et al.*^141^ demonstrated that a nonselective adrenergic α-receptor antagonist, phentolamine, reversed amphetamine-induced gating deficits in rats by increasing S1 and decreasing S2 amplitudes. The β-blocker timolol, in contrast, improved gating by only decreasing S2 amplitude.^141^ A later study in unmedicated male rats demonstrated that α-receptor-mediated effects may be subtype dependent, as in contrast to phentolamine, the α_2_-subtype-specific antagonist yohimbine (0.14 mg kg^−1^) impaired gating.^142^

In support of the translational utility of auditory gating, results from these studies in rodents are predictive of adrenergic effects in humans. Akin to its effect in rats, impaired P50 gating was observed after yohimbine administration (0.40 mg kg^−1^) in healthy human subjects.^143^ Furthermore, Oranje and Glenthoj^144^ have recently reported improved gating after acute administration of clonidine, a selective, anxiolytic α_2-_agonist. Clonidine, however, has undesirable sedative effects due to its high affinity for all α2-receptor subtypes and ability to decrease levels of NE in the brain.^145^ An alternative treatment worthy of future investigation may be guanfacine, an α2A-receptor specific agonist that is 10 × less effective at reducing NE release.^145^

### Nicotinic α7-positive allosteric modulators

A property inherent to all nicotinic receptors is their tendency to desensitize after sufficiently long periods of activation, preventing calcium influx-induced cytotoxicity during prolonged channel opening.^146, 147^ This characteristic may explain why nicotine only transiently improves P50 gating^47, 147^ and is a major concern for clinical trials that examine the effects of α7-receptor agonists.^148^ An alternative strategy is to develop drugs that potentiate agonist activity at sites distinct from the primary active (orthosteric) site. By definition, these compounds are known as allosteric (from the Greek *allos stereos* ‘other solid') modulators. Two primary types of modulators exist. Type I positive allosteric modulators (PAMs) potentiate peak current while preserving desensitization, whereas type II PAMs potentiate peak current, evoke a weak secondary current and reactivate desensitized currents. Type II nicotinic PAMs therefore have received the most interest due to their ability to reduce desensitization. An optimal PAM, however, must also not potentiate channel opening to the extent that it becomes cytotoxic.

Several type II PAMs of the α7-nicotinic receptor have been developed and are currently being investigated in schizophrenia and its associated neurophysiological endophenotypes, such as P50 gating. These compounds have demonstrated efficacy in both animal models of the illness and human patients. The first α7-PAM to be tested for gating effects was 1-(5-chloro-2,4-dimethoxy-phenyl)-3-(5-methyl-isoxazol-3-yl)-urea (PNU-120596). In this study, 0.14 mg kg^−1^ of PNU-120596 significantly reduced amphetamine-induced hippocampal gating deficits in anesthetized rats.^149^ The drug was later shown to be cytotoxic, however, rendering it clinically unfeasible.^150^ A later study using a less toxic PAM ((*N*-(4-chlorophenyl)-α-(((4-chloro-phenyl)amino)methylene)-3-methyl-5-isoxazoleacet-amide), also known as compound 6 or CCMI), found that 0.025 mg kg^−1^ dose of CCMI was sufficient to improve gating in DBA/2 mice.^150^ Another promising compound that has demonstrated preclinical efficacy on sensory gating is 2-((4-fluoro-3-(trifluoromethyl)phenyl)amino)-4-(4-pyridinyl)-5-thiazolemethanol (JNJ-1930942). Similar to other type II PAMs, this compound increases peak current and reduces desensitization. It does not, however, evoke a weak secondary current.^151^

To our knowledge, none of the PAMs described above have yet been clinically evaluated in schizophrenia patients, highlighting the need for additional research in this area. A different α7-PAM, JNJ-39393406, recently showed no significant effects on gating (or any other electrophysiological measure of interest) in patients^152^ despite improving gating in DBA/2 mice (unpublished data cited by Winterer *et al.*). The discrepancy between human and animal findings may be due to differences in α7-receptor expression between patients (~50% loss of receptors in hippocampal CA3 on average)^94^ and DBA/mice (~35% loss of receptors).^42^ It is possible that a more efficacious future treatment strategy will be to use α7-PAMs in subgroups of patients that have relatively preserved levels of receptor expression as determined by positron emission tomography.^153^

## Methodological effects and considerations

### Mental status

One concern when comparing results of animal and human studies on gating paradigms is the effect of anesthesia. The majority of rodent studies examine gating while the animal is anesthetized with a high concentration of choral hydrate, whereas human studies are performed while subjects are awake.

Qualitative examination of the results from studies presented in this review suggests that anesthesia had no effect on the ability of drug effects in animal studies to predict results in patients. It remains possible, however, that anesthesia may affect efficacy depending on the drug tested. Clozapine, for example, has been shown to interact with choral hydrate to reduce activity in serotonergic raphe neurons to a greater extent than either drug alone.^154^ Haloperidol may have differential effects on the inactivation of dopamine neurons depending on whether the animal is anesthetized or awake.^155^ Nicotine's effects of blood flow in the brain may also be differentially modulated depending on the type of anesthesia used.^156^ Overall, however, rodent hippocampal gating appears to be highly predictive of treatment effects in the clinic regardless of mental state.

### Treatment duration

As illustrated in Table 1, the majority animal studies examined the effects of acute doses of drug on gating. In contrast, most human studies have assessed effects after chronic (>6 consecutive days) dosing. Treatment duration did not appear to affect translatability; a drug that improved gating after an acute dose in animals also improved gating at a similar chronic dose in patients. Furthermore, the few animal studies that examined drug effects after acute and chronic dosing demonstrated similar results (for example, DMXB-A studies).

Lack of a treatment duration effect on drug-induced gating improvement may be due to the possibility that sensory gating is an elementary neuronal phenomenon that consequently may be expected to show relatively time-independent dose–response relationships. Unlike psychiatric symptoms, which manifest as the result of countless perturbations in the temporal and spatial network dynamics of complex systems, gating dysfunction is hypothesized to arise in part from abnormalities within a simple neuronal circuit. It is for this reason that clinical trials, for which clinical symptomology is the primary end point, typically examine the effects of chronic administration. In contrast, proof-of-concept and ‘basic' research studies for which a neurophysiological marker (for example, P50 gating) is the primary end point usually examine acute effects.

### Route of drug administration

The goal of this review was to compare the effects of drugs on animal models and schizophrenia patients on gating across similar dose ranges. A limitation of this approach, however, is that the actual ‘dose' of a drug is not only dependent on the amount given but also the route by which it is administered. Indeed, administration route may affect both the rate and extent to which a drug is absorbed, potentially introducing confounding effects due to differences in receptor activation and desensitization.

The majority of animal studies have used intraperitoneal or subcutaneous dose routes, whereas patient studies typically administer drugs orally. Importantly, route of administration does not appear to significantly affect dose–response relationships, as similar doses have comparable effects on gating via consistent mechanisms (for example, ↑S1 and/or ↓S2; Table 1a–c). In addition, drugs that have been administered by different routes (for example, DMXB-A, which has been given intraperitoneally, subcutaneously, intravenously and orally) appear to have qualitatively similar effects. To maximize the likelihood that a given dose will show similar effects in patients, however, future animal studies may wish to examine drug effects using a variety of administration routes.

### Sensory gating in other brain areas

Sensory gating is primarily measured in the hippocampus in rodents, in part, due to findings from Bickford-Wimer *et al.*^20^ who showed greater suppression of S2 in the hippocampus relative to other areas in the auditory pathway. Human neuroimaging studies, for the most part, also suggest that the hippocampus is an important generator of P50 gating and its associated deficits in schizophrenia.^21, 22, 23, 157, 159^ These and other studies have shown, however, that additional brain regions are also significant sources of P50 gating in the human brain. Gating generators may include the thalamus,^23, 158, 160^ superior temporal gyrus/auditory cortex,^161, 162, 163, 164, 165, 166^ medial frontal cortex,^22, 162, 163, 167, 168^ dorsolateral prefrontal cortex,^21, 158, 159, 169^ ventrolateral prefrontal cortex^160^ and insula.^22, 160, 161^ Animal studies have found additional gating generators in the medial septum,^170^ thalamus,^64^ striatum,^171^ amygdala^172^ and medial prefrontal cortex.^173, 174, 175^ How gating in these areas is disrupted in animal models of schizophrenia as well as their ability to predict drug response in patients are important areas for future research.

### Gating of other (mid-latency) potentials

Although the P50 is the most frequently examined potential using paired-stimulus paradigms in schizophrenia, abnormal gating at other potentials has also been observed (for example, the N100 and P200).^176, 177, 178^ These ‘mid-latency' potentials represent later stages of information processing than the P50, and a complete understanding of sensory processing dysfunction in schizophrenia requires thorough examination of how these waveforms are affected in the illness. It is unclear, however, whether the hippocampus has a role in these processes. For example, human electroencephalography studies have primarily localized N100 gating generators to the auditory cortex and association cortices.^179, 180^ Nonetheless, hippocampal contributions to N100 gating cannot be ruled out until other techniques that have greater subcortical spatial resolution (for example, functional magnetic resonance imaging) are used. As functional magnetic resonance imaging by itself has insufficient temporal resolution to capture the N100 potential, a combined electroencephalography/functional magnetic resonance imaging approach may be necessary to more accurately noninvasively assess the role of the hippocampus in the gating of this and other mid-latency-evoked potentials. Once a hippocampal role in N100 gating in humans has been established, animal studies using hippocampal recordings may definitively assess its pharmacological translatability.

## Conclusion

Development of translational assays that predict drug response across species is a priority for psychiatry research. Here, we show that drug effects on auditory P20–N40 gating in rodents as measured from hippocampal electrodes effectively predict effects on P50 gating in schizophrenia patients. To our knowledge, unfortunately, pharmaceutical companies that use sensory gating as a translational screening tool in schizophrenia research are currently in the minority. To that end, this review supports expanded use of sensory gating to increase the probability of success of investigational compounds in therapeutic development.

## Figures and Tables

**Table 1:** **a–c** Summary of drug effects in auditory gating studies, with a focus on schizophrenia

*Drug (primary* *mechanism(s))*	*Species (model)* *or subject*	*Status*	*Dosing*	*Dose(s)* *tested*^a^	*S1, S2* *effects by dose*	*Author* *(year)*
*1a. Nicotinic-based treatments*						
Nicotine (nAChR α4β2 and α7 agonist)	Mouse (DBA/2)	Anesthetized	Acute s.c.	**0.086**	↑S1	Stevens and Wear^43^
	Mouse (C3H with cocaine)	Anesthetized	Acute s.c.	**0.13**	↑S1, ↓S2	Stevens *et al.*^44^
	Mouse (C57Bl/6J with bupropion+Haldol)	Awake	Acute i.p.	**0.083**	↓S2	Siegel *et al.*^35^
	Mouse (DBA/2)	Awake	Acute i.p.	0.0083, 0.025, **0.083**	↑S1 (N40)	Radek *et al.*^45^
	Mouse (C56BL/6J and DBA/2Hsd)	Awake	Chronic s.c.	**0.35 per day**+**0.088** (once per week)	↑S1 (P20), ↓S1 (N40)	Metzger *et al.*^46^
	SZ patients	Awake	Chronic (cigarette)	**0.052** (reverses after 30 m deprivation)	↓S2	Adler *et al.*^47^
	SZ patients	Awake	Chronic (cigarette)	Unspecified (chronic smokers, nicotine deprived >1 h)	No effects	Hong *et al.*^48^
	SZ patients	Awake	Chronic (cigarette)	Unspecified (chronic smokers, nicotine deprived >30 m)	No effects	Olincy *et al.*^49^
Varenicline (nAChR α7 agonist, α4β2 partial agonist)	Mouse (DBA/2)	Anesthetized	Acute i.p.	0.005, **0.025, 0.05, 0.25, 0.5**	↓S2 (0.05), ↑S1 (0.25, 0.5), ↑S1 (trend, 0.025)	Wildeboer-Andrud and Stevens^50^
	SZ patients	Awake	Acute	0.013	No effects	Waldo *et al.*^51^
	SZ patients	Awake	Chronic	**0.013**	↓S2	Hong *et al.*^52^
DMXB-A/GTS-21 (nAChR α7 partial agonist, α4β2 antagonist)	Mouse (DBA/2)	Anesthetized	Acute s.c.	0.028, **0.083, 0.28, 0.83**	0.083, 0.28, 0.83: ↓S2	Stevens *et al.*^53^
	Mouse (C3H with cocaine)	Anesthetized	Acute s.c.	**0.28**	↓S2	Stevens *et al.*^44^
	Mouse (DBA/2)	Anesthetized	Acute oral	0.083, 0.28, **0.83,** 2.75	0.28: ↓S2	Simosky *et al.*^26^
	Mouse (DBA/2)	Anesthetized	Chronic s.c. (minipump)	**0.015, 0.03,** 0.06 per day	0.015, 0.03, 0.06 per day: ↓S2	Stevens *et al.*^54^
	SZ patients	Awake	Acute	**0.95,** 1.89	0.95: ↓S2	Olincy *et al.*^25^
Tropisetron (α7 partial agonist, 5- HT(3) antagonist)	Mouse (DBA/2)	Anesthetized	Acute i.p.	0.025, **0.083, 0.25**	↑S1, ↓S2 (analysis conducted for 0.083 only)	Hashimoto *et al.*^55^
	SZ patients	Awake	Acute	**0.13**	No significant effects (↑gating driven by NS ↑S1, ↓S2)	Koike *et al.*^56^
	SZ patients	Awake	Chronic	**0.13**	S1, S2 data not reported	Shiina *et al.*^57^
	SZ patients	Awake	Chronic	**0.063, 0.13, 0.25**	↑S1 (0.13, 0.25), ↓S2 (0.063–0.25)	Zhang *et al.*^58^
Donepezil (acetylcholinesterase inhibitor)	Rat (unmedicated)	Awake	Acute oral	**0.43**	↑S1, ↓S2	Klinkenberg *et al.*^59^
	SZ patients	Awake	Chronic	0.065 per day for 4 weeks, 0.13 per day for 2 weeks	None	Buchanan *et al.*^60^
Perinatal choline (ACh precursor, α7 agonist)	Mouse (DBA/2)	Anesthetized	Chronic diet	**5** × **normal diet**	↓S2	Stevens *et al.*^61^
	Mouse (CHRNA7 WT, het, null)	Anesthetized	Chronic diet	**5** × **normal diet (WT only)**	↓S2 (WT only)	Stevens *et al.*^62^
	Human infants	Sleeping	Chronic	**2** × **normal diet**	No significant effects (↑gating driven by NS ↓S2)	Ross *et al.*^63^

*1b. Dopaminergic-based treatments*						
Haldol and/or other typical antipsychotics (D2 antagonists)	Rat (amphetamine)	Anesthetized	Acute i.p.	**0.14**	Significance level not reported	Bickford-Wimer *et al.*^20^
	Rat (amphetamine)	Anesthetized	Acute i.v.	**0.043**	Significance level not reported	Krause *et al.*^64^
	Mouse (DBA/2)	Anesthetized	Acute i.p.	0.083	↑S2	Simosky *et al.*^65^
	Mouse (bupropion)	Awake	Chronic	0.083	↑S1, ↑S2	Siegel *et al.*^35^
	SZ patients	Awake	Chronic	0.14	↑S1, ↑S2	Freedman *et al.*^66^
	SZ patients	Awake	Chronic	0.063–0.38	↑S1; S2 significance level not reported	Adler *et al.*^67^
	SZ patients	Awake	Chronic	0.19	No effects	Arango *et al.*^68^
	SZ patients	Awake	Chronic	0.13	No effects	Sanchez-Morla *et al.*^69^
	SZ patients	Awake	Chronic	0.073 (Haldol equivalent of amisulpride)	No effects	Düring *et al.*^70^

*1c. Serotonergic-based treatments*						
Ondansetron (5-HT(3) antagonist)	Mouse (DBA/2)	Anesthetized	Acute i.p.	0.0083, **0.028, 0.083**, 0.25	↑S1 (0.028, 0.083), ↓S2 (0.028 (trend), 0.083, 0.25)	Wildeboer *et al.*^71^
	SZ patients	Awake	Acute	**0.20**	↓S2	Adler *et al.*^72^
Olanzapine (5-HT(2) and D2 antagonist)	Mouse (DBA/2)	Anesthetized	Acute i.p.	**0.00083, 0.0028, 0.0083, 0.028**	↑S1 (8.3E^−^^4^, 0.028), ↓S2 (0.0028)	Simosky *et al.*^73^
	SZ patients	Awake	Chronic	**Unspecified**^b^ (effects grouped with those of other atypicals)	Significance level not reported	Light *et al.*^133^
	SZ patients	Awake	Chronic	0.19	No effects	Arango *et al.*^68^
	SZ patients	Awake	Chronic	Unspecified^b^	↓S1	Adler *et al.*^74^
	SZ patients	Awake	Chronic	0.30	No effects	Sanchez-Morla *et al.*^69^
Clozapine (5-HT(2A), 5-HT(3) and D4 antagonist)	Mouse (DBA/2)	Anesthetized	Acute (i.p.)	**0.0083, 0.0083, 0.28, 0.83**	0.0083–0.28: ↓S2; 0.83: ↑S1	Simosky *et al.*^65^
	Rat (amphetamine)	Anesthetized	Acute (i.p.) and chronic (oral)	Acute: 0.71 (trend to improve) Chronic: 9.28 per day	Acute 0.71: ↑S1; Chronic 9.28 per day: none	Joy *et al.*^75^
	Mouse (DBA/2)	Anesthetized	Acute (i.c.v.)	0.1, **0.5, 1** (μg per mouse)	0.5: ↑S1 (trend), ↓S2; 1: ↑S1, ↓S2	Abrams *et al.*^76^
	Mouse (DBA/2)	Anesthetized	Chronic i.c.v. (once a day or continuous)	Daily: **3, 7.5, 15, 30** (μg per day per mouse) Continuous: 3, 7.5, **15, 30** (μg per day per mouse)	Daily 15, 30 μg per day: ↑S1; Continuous 15 μg per day: ↓S2; Continuous 30 μg per day: ↑S1, ↓S2	Stevens *et al.*^77^
	SZ patients	Awake	Chronic	**3.68**	↑S1	Nagamoto *et al.*^78^
	SZ patients	Awake	Chronic	**3.67**	↑S1	Nagamoto *et al.*^79^
	SZ patients	Awake	Chronic	**Unspecified**^b^ (effects grouped with those of other atypicals)	Significance level not reported	Light *et al.*^133^
	SZ patients	Awake	Chronic	**Unspecified**^b^	↑S1	Becker *et al.*^80^
	SZ patients	Awake	Chronic	**Unspecified**^b^	↓S2	Adler *et al.*^74^
	SZ patients	Awake	Chronic	3.40	None	Hong *et al.*^81^
	SZ patients	Awake	Chronic	6.02	None	Sanchez-Morla *et al.*^69^

Abbreviations: 5-HT, serotonin receptor; ACh, acetylcholine; CHRNA7, nicotinic α7 receptor gene; CPZ, chlorpromazine; D2R, dopamine D2 receptor; DMXB-A, 3-2,4 dimethoxybenzylidene anabaseine; het, heterozygote; i.c.v., intracerebroventricular; i.p., intraperitoneal; i.v., intravenous; nAChR, nicotinic receptor; NS, nonsignificant; PAM, positive allosteric modulator; S1, stimulus 1; S2, stimulus 2; s.c., subcutaneous; SZ, schizophrenia; WT, wild type.

a Doses were corrected for species' surface area (1/12 correction for mice, 1/7 correction for rats);^82^ human doses assume body weight of 175 lbs; doses in mg kg^−1^ unless specified. Doses that significantly improved gating are in bold.

b Unspecified doses were most likely in the therapeutic range for each medication (clozapine, 2–10 mg kg^−1^; olanzapine, 0.06–0.25 mg kg^−1^).

‘Chronic' is defined as greater than six consecutive days of administration. ‘Effective doses' and S1 and S2 effects are *P*<0.05 unless specified. ‘Trend' level is defined as 0.05<*P*<0.10. Human dosing is oral unless specified.

## Box 1 Rodent models of schizophrenia that show impaired hippocampal sensory gating

*α7 Mutants*. Schizophrenia is associated with reduced expression of hippocampal α7 nicotinic receptors that is hypothesized to contribute to P50 gating deficits. To model this endophenotype, mutant mice have been bred to express reduced levels of α7 protein. Accordingly, null mutant mice express no α7 protein, and het mice express 50% of normal levels. The het mice most closely match schizophrenia patients in regard to expression level.^27, 28^

*Psychostimulants.* Psychostimulant-based animal models of schizophrenia have been primarily developed based on the dopamine hypothesis of the illness, which posits that the disease is caused by hyperactivity of mesolimbic dopamine neurons in the brain. Accordingly, stimulants such as amphetamine and cocaine induce a rapid and robust increase in dopamine release in these neurons (first reviewed by Moore (1977)^29^). In support of the model, psychostimulant administration induces psychosis in healthy human subjects^30, 31, 32, 33^ as well as ‘positive symptom'-associated behaviors in animals, such as hyperlocomotion and stereotypy.^34^

*Bupropion*. Bupropion is an antidepressant that has been used to model gating deficits in mice due to its function as a dopamine, norepinephrine and serotonin reuptake inhibitor as well as a nicotinic receptor antagonist. These receptor actions may pharmacologically mimic some aspects of neurochemical dysfunction in schizophrenia.^35^

*DBA/2 mouse*. The most commonly used rodent model for gating studies, the DBA/2 mouse effectively recapitulates a number of symptoms of schizophrenia, including learning and memory deficits,^36, 37^ attention deficits^38, 39^ and abnormal social behavior.^40, 41^ Interestingly, DBA/2 mice also show 35% reduced expression of α7 receptors relative to C3H wild-type strains.^42^
